# Current status and influencing factors of activation of older patients with chronic disease

**DOI:** 10.3389/fpubh.2023.1308196

**Published:** 2024-01-24

**Authors:** Zhu Yiran, Zhang Yan, Xing Lijun, Li Xizheng, Zhang Xinjie, Tian Yutong

**Affiliations:** School of Nursing and Health, Zhengzhou University, Zhengzhou, Henan, China

**Keywords:** chronic disease, patient activation, health empowerment, e-health literacy, cross-sectional study

## Abstract

**Objective:**

We aimed to investigate the status and influencing factors of activation of older patients with chronic disease.

**Methods:**

We conducted a cross-sectional study, using the general information questionnaire, Patient Activation Measure, the Chinese version of the e-Health Literacy Scale, and the Health Empowerment Scale for the Elderly with Chronic Disease. By the convenience sampling method, 289 older patients with chronic disease were selected from January to April 2023 in a Class A tertiary hospital in Zhengzhou.

**Results:**

The mean score of the Patient Activation Measure for older patients with chronic disease was 65.94 ± 13.35. The association of influencing factors such as religion, family income, health empowerment, e-health literacy, and patient activation was investigated.

**Conclusion:**

The patient activation of older patients with chronic disease was at a middle level. Patients without religion and from high-income families tended to have a higher level of patient activation. Improving health empowerment and e-health literacy levels promotes patient activation and enhances their self-health management ability.

## 1 Introduction

Patient activation (PA), mainly for patients with chronic diseases, was first proposed by Hibbard et al. (1) in the United States, which refers to the level of patients to participate initiatively in healthcare. It is manifested in the fact that the patient values his or her own role and position during the management of the chronic disease, possesses self-health management ability as well as the knowledge, skills, confidence, and behaviors for preventing the deterioration of the disease, actively seeks for health-related information and help from medical personnel, and can adhere to perform health-related behaviors (2). Currently, the prevalence of chronic diseases among the older adults in China reached 59.1%. Among them, the activation level of older hospitalized patients with chronic diseases was low (3), making it difficult for them to be integrated into the chronic disease self-management system. However, the improvement of the enthusiasm level of older hospitalized patients with chronic diseases can help them maintain their strong health management ability after discharge. Therefore, improvement programs for patient enthusiasm increasingly receive the attention of scholars. e-health literacy is the ability of people to search, find, understand, and evaluate health information from electronic resources, and then process and apply the information to solve health problems (4). This variable has a positive impact on the self-health management skills of patients with chronic diseases. In addition, health empowerment is a process in which patients actively utilize their knowledge and abilities to enhance their sense of self-efficacy to control their diseases, manage their lives, and promote their health (1). In addition, it is a process to develop the capacity for health promotion, which promotes an individual’s intrinsic confidence, control ability, and the ability to solve problems independently. Meanwhile, it is a predictor of the self-management of chronic disease (5). Thus, the improvement of the level of health empowerment is one of the most important measures to control chronic disease. At present, domestic research on the activation of chronic disease patients mainly focuses on the community or family, and there is less research on older patients hospitalized with chronic diseases. This study aimed to investigate the activation level of older patients hospitalized with chronic diseases and associated influencing factors. This will provide a reference measure to support the development of targeted interventions to improve the activation level of these patients.

## 2 Participants and methods

### 2.1 Study participants

The convenience sampling method was used to select 289 older patients with chronic disease from January to April 2023 in a Class A tertiary hospital (which is the highest level hospital in China according to the provisions of China’s current “Hospital Classification and Management Measures”) in Zhengzhou. Inclusion criteria are as follows: 1) patients aged ≥ 60 years old; 2) clinically diagnosed with chronic disease and admitted to related departments (including endocrinology medicine, cardiovasology medicine, respiratory medicine, gastroenterology medicine, nephrological medicine, and medical oncology); 3) patients in stable condition or soon to be discharged; and 4) voluntary participation in this study. Exclusion criteria are as follows: 1) patients in the end-stage of the disease; 2) patients with significant visual or hearing impairment; and 3) patients with cognitive disorders. This is a cross-sectional study. The sample size was estimated based on the influencing factors concerning patients’ activation of the older adults with chronic disease. According to the empirical method to calculate the sample size, which involved a total of 24 variables in this study, taking 5–10 times the number of variables, and then considering a 10% missed visit rate, the calculated sample size range was 133–267.

### 2.2 Survey tools

#### 2.2.1 General information questionnaire and clinical data

The general information questionnaire was designed by the researchers and included items concerning age, gender, religious affiliation, permanent residence, living arrangement, education level, family *per capita* monthly income, length of stay in the hospital, whether the patient was a multiple chronic disease patient or not, department, etc.

#### 2.2.2 Patient Activation Measure

Patient Activation Measure (PAM) was developed by Hibbard et al. and was translated into Chinese and revised by Chen Shiqiao (6) to assess the knowledge, skills, and confidence of chronic disease patients in self-health management. There are 13 items, and the answering categories per item are 4-point Likert scales, ranging from totally disagree to totally agree with scores from 1 to 4 and “non-applicable” with a score of 0. Calculation: If all entries do not appear not applicable to the answer, then the 13 entries’ scores are directly added to the corresponding original score; ② if there is a choice of “not applicable” option or no answer, the situation should be treated as “missing value,” the original score = (total score of entries other than missing values×13)/(13 – number of entries with missing values); The raw score was converted to a 100-point scale, and the conversion formula is as follows: raw score×100/(13 × 4) = final score (3). In this study, at least 10 entries need to be completed and valid scores need to be obtained so that the total score can be calculated. Activity scores vary from 0 to 100. According to the scores, there are four levels. Level 1, the lowest activity with a score of ≦47.0, refers to the fact that the patient has been passively listening to the arrangement of the medical personnel without realizing the importance of his/her position in health management. Level 2, with scores of 47.1–55.1, refers to the fact that the patient has consciously been responsible for his/her own health management without rich knowledge and nursing experience to support the action. Level 3 with scores of 55.2–67.0 refers to the fact that patients have begun to take actions to improve their health situation, but they did not have enough disease-related skills and confidence to maintain routines. Level 4, the highest activity with scores of >67.1, refers to patients managing their self-health with enough knowledge, skills, and confidence, but they still need external help under stress. Higher scores on the scale mean higher patient activation. The overall Cronbach’s α coefficient of the PAM is 0.82–0.87.

#### 2.2.3 The Chinese version of the e-Health Literacy Scale

The e-Health Literacy Scale was developed by Norman et al. (7) and was translated into Chinese and revised by Guo Shuaijun et al. (8). The scale contains three dimensions and eight items, which are divided into e-health and services application skills (five items), judgment skills (two items), and decision-making skills (one item). The answering categories per item are based on a 5-point Likert scale ranging from totally disconformit to totally conformit with a total score range of 8–40. Divided by 26 as the boundary value, with ≥26 being a high e-health literacy level and < 26 being a low e-health literacy level. Higher scores reflected a high level of e-health literacy. The overall Cronbach′s α coefficient is 0.913.

#### 2.2.4 The health empowerment scale for older adults with chronic disease

This scale was developed by Yang et al. (9). The scale contains 5 dimensions and 26 items, which are divided into beliefs of responsibility (4 items), obtaining support (5 items), enriching knowledge (5 items), participation in treatment (6 items), and reconstructing the self (6 items). The answering categories per item are based on a 5-point Likert scale ranging from totally disagree to totally agree with scores from 1 to 5. Health empowerment scores vary from 26 to 130. Higher scores reflected a high level of health empowerment. The overall Cronbach′s *α* coefficient of this scale was 0.927.

### 2.3 Data collection

The study was a cross-sectional survey study. Professionally trained researchers distributed questionnaires to the patients on the spot and explained the content, the purpose of the study, and how to complete the questionnaire with uniform instructional language. After obtaining the patients’ consent, patients were asked to finish the questionnaires by themselves according to their own conditions to ensure authenticity and validity. For patients who could not complete the questionnaire independently, the researcher provided explanations and then assisted them in completing it. All the data were checked and collected on the spot after completion. If any issues regarding its content arose, the researcher would immediately return it to the participant after the latter had completed it for correction.

### 2.4 Statistical methods

The SPSS Statistics 26.0 software program was used for data entry and analysis. Count data were expressed as rate or composition, and measurement data were expressed as median and interquartile after the normality test using Student’s *t*-test, one-way analysis of variance (ANOVA), or H-test to analyze between two groups. The correlation between health empowerment, e-health literacy, and patient activation was analyzed using Pearson’s correlation. Taking the total score of patient activation as the dependent variable, the variables with statistical significance in the univariate and correlation analyzes were included as independent variables in the multiple linear regression equation; then, the multiple linear regression analysis was carried out, and *p* < 0.05 was considered to be statistically significant.

## 3 Results

### 3.1 Participants’ characteristics

A total of 312 questionnaires were subsequently distributed, 23 invalid questionnaires were excluded (19 incomplete questionnaires and 4 multiple answers), and 289 valid questionnaires were recovered (effective response rate, 92.63%). The participants’ demographic characteristics are presented in Table 1.

**Table 1:** Univariate analysis of motivation scores of older chronic disease inpatients (*n* = 289).

Characteristics	Number of cases (composition ratio) [*n* (*%*)]	Score ( $\bar{x}$ *±s*)	*t*/F/H value	*p*-value
*Sex*			1.714^a^	0.088
Male	107 (37.02)	67.69 ± 14.09		
Female	182 (62.98)	64.92 ± 12.83		
*Age/Years*			1.945^a^	0.053
<65	136 (47.06)	67.56 ± 12.42		
≧65	153 (52.94)	64.51 ± 14.01		
*Religious affiliation*			−3.512^a^	0.001
Yes	48 (16.61)	59.88 ± 13.26		
No	241 (83.39)	67.15 ± 13.06		
*Permanent residence*			17.624^c^	<0.001
Countryside	114 (39.45)	62.91 ± 10.43		
Cities and towns	89 (30.80)	64.94 ± 15.22		
Municipalities	86 (29.76)	71.00 ± 13.42		
*Living arrangement*			9.139^b^	<0.001
Living alone (or in an institution)	8 (2.77)	85.26 ± 6.72		
Live together as husband and wife	173 (59.87)	65.58 ± 13.39		
Cohabitation with children	108 (37.37)	65.10 ± 12.63		
*Number of children*			−2.341^a^	0.020
≦1	74 (25.60)	62.83 ± 14.45		
>1	215 (74.39)	67.01 ± 12.81		
*Education level*			26.817^c^	<0.001
College and above	36 (12.46)	75.93 ± 13.52		
High school/technical school/secondary school	57 (19.72)	67.90 ± 15.44		
Junior high school	81 (28.03)	64.13 ± 12.39		
Primary and below	115 (39.79)	63.13 ± 11.21		
*Previous occupation*			33.105^c^	<0.001
Worker	46 (15.92)	63.85 ± 14.53		
Farmer	123 (42.56)	61.97 ± 9.76		
Medical worker	8 (2.77)	75.48 ± 14.35		
Teacher	22 (7.61)	71.80 ± 12.37		
Civil servants or business employees	47 (16.26)	72.33 ± 12.69		
Else	43 (14.88)	67.79 ± 17.23		
*Family per capita monthly income/Yuan*			11.184^c^	0.004
<1,000	50 (17.30)	63.53 ± 9.38		
1,000 ~ 3,000	101 (34.95)	63.54 ± 13.82		
>3,000	138 (47.75)	68.57 ± 13.80		
*Longest duration of a chronic disease/Year*			0.559^b^	0.692
<1	92 (31.83)	66.48 ± 12.55		
1 to 5	104 (35.99)	65.76 ± 14.34		
6 to 10	45 (15.57)	63.67 ± 11.90		
>10	48 (16.60)	67.44 ± 14.01		
*Length of stay in hospital / Day*			6.204^c^	0.102
Multi-treatment patients	11 (3.81)	72.93 ± 10.97		
3–7	127 (43.94)	64.85 ± 12.70		
8–14	105 (36.33)	66.37 ± 14.17		
>14	44 (15.22)	67.11 ± 13.26		
*Accompanied or unaccompanied during hospitalization*			5.378^b^	0.005
Always	75 (25.95)	62.27 ± 12.76		
Occasional	193 (66.78)	66.73 ± 13.20		
Never	21 (7.27)	71.85 ± 14.12		
*Whether or not they initiate discussions with patients about their condition*			56.323^c^	<0.001
Volunteer	145 (50.17)	71.15 ± 11.26		
Not initiated, but will participate	105 (36.33)	63.06 ± 12.59		
Non-participation	39 (13.49)	54.34 ± 13.13		
*Whether you have multiple chronic diseases*			0.192^a^	0.848
Yes	177 (61.25)	66.06 ± 13.53		
No	112 (38.75)	65.75 ± 13.11		
*Medical payment methods*			42.496^c^	<0.001
Urban workers’ medical insurance	57 (19.72)	74.15 ± 12.50		
Medical insurance for urban and rural residents	46 (15.92)	67.56 ± 15.11		
NACS	142 (49.13)	64.23 ± 11.56		
Else	44 (15.22)	58.89 ± 12.31		
*Department*			2.103^c^	0.835
Nephrology	51 (17.65)	64.66 ± 12.80		
Cardiology	40 (13.84)	65.99 ± 14.57		
Oncology	57 (19.72)	66.61 ± 14.21		
Endocrinology	49 (16.56)	67.43 ± 13.50		
Respiratory medicine	44 (15.22)	64.28 ± 12.75		
Gastroenterology	48 (16.61)	66.49 ± 12.61		

a is *t*-value; b is F-value; c is H-value.

### 3.2 The patient activation, health empowerment, and e-Health Literacy Scores

According to the Hibbard exclusion criteria for dividing the total patient activation score (percentage), the mean patient activation score of the 289 older patients with chronic diseases in this study was 65.94 ± 13.35. Details of the scores for each dimension are shown in Table 2.

**Table 2:** Scores on Each dimension of activation, health empowerment, and e-Health Literacy Scale ( $\bar{x}$ *±s*, score).

Scale classification	Dimensional ordering	Dimension name	Mean score of entries in the dimension
Patient Activation Scale	1	Cognitive dimension	2.99 ± 0.71
	2	Belief dimension	2.62 ± 0.70
	3	Skill dimension	2.59 ± 0.57
	4	Action dimension	2.47 ± 0.65
Chinese version of e-Health Literacy Scale		Totals	2.13 (1.50, 3.13)
	1	E-health and services application skills	2.20 (1.40, 3.20)
	2	Judgment skills	2.00 (1.00, 3.50)
	3	Decision-making skills	2.00 (1.00, 3.00)
Health empowerment scale for older adults with chronic disease		Totals	3.23 (2.52, 3.81)
	1	Beliefs of responsibility	3.50 (2.50, 4.25)
	2	Obtaining support	3.40 (2.80, 3.90)
	3	Reconstruct the self	3.17 (2.67, 4.00)
	4	Participation in treatment	3.17 (2.17, 3.83)
	5	Enriching knowledge	3.00 (2.20, 3.80)

In the 13 items of the Patient Activation Scale, the three lowest scores were, in order, “I know that I have different treatment options for my health” (2.21 ± 0.84 points), “I understand the source of the problems and origins of my health problems” (2.26 ± 0.82 points), and “I know how to avoid making my health condition more problems” (2.43 ± 0.80 points). Details of the ranking of the scores of the respective items from highest to lowest are shown in Table 3.

**Table 3:** Enumeration of items with the lowest scores on the patient activation, health empowerment, and e-Health Literacy Scales ( $\bar{x}$ *±*s, Scores).

Scale	Content of items	Affiliated dimensions	Score
Patient Activation Scale	I know that every prescription drug is prescribed by a physician	Skill dimension	2.46 ± 0.84
	I am confident that I will find a solution to my health issues when I confront new situations and problems	Belief dimension	2.46 ± 0.85
	I know how to prevent further problems with my health condition	Action dimension	2.43 ± 0.80
	I understand the source of the problems and origins of my health problems	Skill dimension	2.26 ± 0.82
	I know that I have different treatment options for my health	Action dimension	2.21 ± 0.84
Health empowerment scale for older adults with chronic disease	I participate in the development of treatment programs	Participation in treatment	2.71 ± 1.19
	I am active in group activities	Obtaining support	2.62 ± 1.25
	I know what the test results mean	Enriching knowledge	2.59 ± 1.29
Chinese version of e-Health Literacy Scale	I feel confident in using information from the Internet to make health decisions	Decision-making skills	2.35 ± 1.25
	I know how to use the internet to answer my health questions	Judgment skills	2.34 ± 1.28
	I am able to differentiate between high- and low-quality health information on the Internet	e-health and services application skills	2.33 ± 1.27

### 3.3 Influencing factors of patient activation with chronic diseases

#### 3.3.1 Univariate analysis of patient activation in older chronic disease patients

The results of the univariate analysis (Table 1) showed that there were significant differences in patient activation scores among the older adults with whether they follow a religion or not, have permanent residence, have a living arrangement, have children, are educated or not level, had a previous occupation, have family *per capita* monthly income, and were accompanied or unaccompanied during hospitalization, whether they initiate discussions with patients about their condition or not, medical payment methods (*p* < 0.05).

#### 3.3.2 Correlations between health empowerment, e-health literacy, and patient activation in older chronic disease patients

As shown in Table 4, the results of Pearson’s correlation analysis showed that the total PAM score of older patients with chronic disease positively correlated with the total score of the e-Health Literacy Scale, E-health and services application skills, judgment skills, and decision-making skills (*r* = 0.618, 0.585, 0.521, 0.575, both *p* < 0.05) and that the total PAM score of older patients with chronic disease positively correlated with total score of the Health Empowerment Scale, beliefs of responsibility, obtaining support, enriching knowledge, participation in treatment, and reconstructing the self (*r* = 0.940, 0.768, 0.827, 0.834, 0.764, 0.768, both *p* < 0.05).

**Table 4:** Correlation between e-health literacy, health empowerment, and patient motivation (*n* = 289).

Items		Correlation coefficient
Chinese version of e-Health Literacy Scale	Totals	0.618^*^
	E-health and services application skills	0.585^*^
	Judgment skills	0.521^*^
	Decision-making skills	0.575^*^
Health empowerment scale for older adults with chronic disease	Totals	0.940^*^
	Beliefs of responsibility	0.768^*^
	Obtaining support	0.827^*^
	Enriching knowledge	0.834^*^
	Participation in treatment	0.764^*^
	Reconstruct the self	0.768^*^

*p<0.05.

#### 3.3.3 Multiple stepwise linear regression analysis of the factors influencing patient activation in older chronic disease patients

The scores of patient activation in older chronic disease patients were used as the dependent variable, and 12 statistically significant variables in the univariate analysis and correlation analysis were included as the independent variables for multiple stepwise linear regression analysis. The value assignment of independent variables is shown in Table 5. The regression results [in Table 6] showed that “Whether they have religion,” “family *per capita* monthly income,” health empowerment, and e-health literacy were factors influencing patient activation of older chronic disease patients.

**Table 5:** Assignment methods of independent variables.

Considerations	Variable name		Description of the assignment
Religious affiliation	X_1_		是 = 1, 否 = 0
Permanent residence	X_2_		countryside = 1, cities and towns = 2, municipalities = 3
Living arrangement	X_3_		Set dummy variable
		Living alone or in an institutionX_31_	X_31_ = 1 ×_32_ = 0
		live together as husband and wifeX_32_	X_31_ = 0 ×_32_ = 1
		Cohabitation with childrenX_33_	X_31_ = 0 ×_32_ = 0 (Reference)
Number of children	X_4_		≦1 = 1, >1 = 2
Education level	X_5_		College and above = 1, High school/technical school/secondary school = 2, Junior high school = 3, Primary and below = 4
Previous occupation	X_6_		Set dummy variable
		WorkerX_61_	X_61_ = 1 ×_62_ = 0 ×_63_ = 0 ×_64_ = 0 ×_65_ = 0
		FarmerX_62_	X_61_ = 0 ×_62_ = 1 ×_63_ = 0 ×_64_ = 0 ×_65_ = 0
		Medical workerX_63_	X_61_ = 0 ×_62_ = 0 ×_63_ = 1 ×_64_ = 0 ×_65_ = 0
		TeacherX_64_	X_61_ = 0 ×_62_ = 0 ×_63_ = 0 ×_64_ = 1 ×_65_ = s0
		Civil servants or business employeesX_65_	X_61_ = 0 ×_62_ = 0 ×_63_ = 0 ×_64_ = 0 ×_65_ = 1
		ElseX_66_	X_61_ = 1 ×_62_ = 0 ×_63_ = 0 ×_64_ = 0 ×_65_ = 0 (Reference)
Family *per capita* monthly income/Yuan	X_7_		<1,000 = 1, 1,001 ~ 3,000 = 2, >3,000 = 3
Accompanied or unaccompanied during hospitalization	X_8_		Always = 1, Occasional = 2, Never = 3
Whether or not they initiate discussions with patients about their condition	X_9_		Initiate discussions = 1, Not initiating, but will participate in discussions = 2, Non-participation in discussions = 3
Medical payment methods	X_10_		Set dummy variable
		Urban workers’ medical insurance X_101_	X_101_ = 1, X_102_ = 0, X_103_ = 0
		Medical insurance for urban and rural residents X_102_	X_101_ = 0, X_102_ = 1, X_103_ = 0
		NACS X_103_	X_101_ = 0, X_102_ = 0, X_103_ = 1
		Else X_104_	X_101_ = 0, X_102_ = 0, X_103_ = 0 (Reference)
Chinese version of e-Health Literacy Scale	X_11_		Input as original value
Health empowerment scale for older adults with chronic disease	X_12_		Input as original value
Patient Activation Scale	Y		Entered as a percentage value

**Table 6:** Multiple stepwise linear regression analysis of factors influencing the activation of 289 older inpatients with chronic diseases.

Variant	Regression coefficient (*B*)	Standard error (*SE*)	Standardized regression coefficients	*t*	*p*
Constant	20.202	3.430		5.889	<0.001
Religious affiliation	1.846	0.850	0.052	2.172	0.031
Family *per capita* monthly income	0.976	0.476	0.055	2.052	0.041
e-Health Literacy Scale	0.194	0.048	0.128	4.058	<0.001
Health empowerment scale	0.494	0.021	0.796	23.893	<0.001

## 4 Discussion

### 4.1 The current status of patient activation in older chronic disease patients

The results showed that the patient activation of 289 older patients with chronic disease was 65.94 ± 13.35. Being at the middle level, this was consistent with the results of the study conducted by Zheng Minjia (10). The analysis of the possible reasons is listed as follows. First, chronic disease has the characteristics of a long course and is difficult to cure, which increases the burden of self-feeling about medical costs and long-term care. Therefore, participants are more likely to be in a state of anxiety, depression, or other negative psychology, which may negatively affect the patient activation of the older adults with chronic disease. Second, influenced by many factors, people with chronic illness need to manage self-health from lifestyle, drug therapy, psychological dredge, and other aspects. However, older chronic disease inpatients found increasing difficulty in self-health management due to the worse grasp of disease knowledge which is affected by their physiology, cognition, and body disease. Third, older chronic disease patients in China have poor consciousness of self-health management (11) and do not wholly catch the connotation of the concept that one should be the first line of defense in his/her own health, resulting in the reduction of patient activation. Another study (12) has shown that chronic disease patients’ activation level directly affects their initiative of response measures for the disease. Strengthening the condition cognitively, enhancing confidence in disease management, and upgrading the skills, they need to raise chronic disease patients’ attention to prolonged treatment. Therefore, multiple measures should be taken by medical personnel to increase knowledge and raise awareness so that patients can actively participate in disease management. At the same time, it is recommended that medical personnel monitor the mental health of older chronic disease inpatients to identify whether there is a negative impact of the chronic disease. Intervening on negative feelings (e.g., anxiety and depression) and raising awareness of each individual responsibility can motivate patient activation.

### 4.2 Analysis of the influencing factors of patient activation in older chronic disease patients

#### 4.2.1 Religious affiliation

The results of this study showed that religion was an independent influencing factor on the level of activation of older patients hospitalized with chronic disease. Compared with the older sdults with no religion, the older sdults with a religion had a lower level of patient activation. Several studies (13, 14) also showed that patients with no religion have a better level of self-management than patients with a religion. Patients with religion tend to have a set of beliefs about life and death and often have independent opinions and understandings on issues related to life and health, so they are misinterpreted and misjudged (13) and believe the development and prognosis of the disease cannot be changed by themselves. On the contrary, patients with no religion are generally more receptive to scientific health knowledge and believe that they can improve their disease control through their own or outside help. It is suggested that the religious background of patients and their caregivers should be fully considered to avoid violating taboos and to meet the needs of patients and their caregivers for religious/spiritual support (15) when caring for hospitalized patients with religion. Family members often have a high degree of homogeneity in religion, which also affects the patient’s attitude toward the disease, so it is recommended for healthcare professionals to conduct disease-related education for older patients hospitalized with chronic disease and their family members to improve their acceptance of the disease, as well as the awareness and participation of self-management, so as to improve the patient’s degree of activation.

#### 4.2.2 Family *per capita* monthly income

The results of this study showed that the more family *per capita* monthly income the older patients get, the higher level of patient activation they have, which is consistent with the conclusion of Ngooi et al. (16). The possible causes are as follows. Because of the chronic disease characteristics of high incidence and lifelong medicine, patients with high family *per capita* monthly income are more likely to have more family financial support so that they would be equipped with the relatively advanced risk-resistant ability to face the disease. Also, they do well in some aspects, such as diet, lifestyle adjustments, regular outpatient follow-up, knowledge and skills of utilizing essential public health services, and so on (17). Meanwhile, people with high income would spend more in investing their health, for example, smartphones, which offer easy access to health resources. However, patients with low family *per capita* monthly income are more likely to be depressed, anxious, and upset due to great physical and psychological pressure, which makes them get a low level of patient activation. This suggests that medical personnel are expected to pay attention to the older inpatients who are hard up financially and to help them and their families gain more social network support and improve the level of patient activation to alleviate the poverty and disability due to illness.

#### 4.2.3 e-health literacy

With the development of digital management of chronic disease, patients with e-health literacy, which serves as an essential skill in the digital age, can leisurely cope with the transition of the medical environment, which eases under-allocation, uneven distribution, and cost of health resources, so e-health literacy plays an important role in improving health outcomes and quality of life of older chronic disease patients. As a matter of fact, the result of this study showed that the e-health literacy degree of 289 older chronic disease inpatients was at a low level, and it is an influencing factor of the patient activation in this research. Analysis of the reasons may be related to the fact that 114 participants in this survey come from villages that lack knowledge and skills in using the Internet, which may serve as a determining factor in hindering them from acquiring health-related messages. In addition, there were 153 senior participants aged more than 65 years whose weakness of physical function also hampers access to electronic health information. The finding of Sheng et al. (18) indicated an interaction between e-health literacy and web information seeking. People with high e-health literacy are more likely to pursue health information and utilize resources Then, they are encouraged to manage self-health better so that they can gain a high level of patient activation. Furthermore, Li et al. (19) found that older people with high e-health literacy are more likely to be with a stronger subjective initiative to adopt health-promoting behaviors. In addition, peer effects play a positive role in promoting the e-health literacy level of the older adults with chronic disease. Accordingly, it is advisable for medical staff to be concerned about e-health literacy, an influencing factor, and manage to create intelligent classes and offer support groups for chronic disease for the older adults and other measures to improve their e-health literacy. Furthermore, medical personnel are supposed to take an active part in developing personalized health information devices for the older chronic disease inpatients, such as adjustment of font size, simplification of the operation process, and so on, in order to continually improve the utilizing capacity level of chronic disease resources to promote the patient activation.

#### 4.2.4 Health empowerment

The results of this study point out that the health empowerment score of the older chronic disease inpatients was at the middle level, which was in line with the finding of Gu Kedong et al. (20), and health empowerment was also proved to be the influencing factor of patient activation. That is probably because people with health empowerment subjectively realize they get more social support, which can give certain hope and a sense of direction while living with a positive state of mood, as a breakthrough can break the cycle of negative self-perception, which can promote patients to recognize their self-worth and then they would take a positive approach to respond to disease, and then actively search health information (21) and gain higher patient activation consequently. In conclusion, health empowerment can raise the self-awareness of decision-making and gradually strengthen the confidence in controlling the disease and then improve the patient activation of older chronic disease patients. Furthermore, a positive perception of patients can heighten their self-efficacy in dealing with disease and encourage them to actively participate in the management of disease, which makes them get high patient activation as a consequence. However, how can the process of health empowerment influence patients’ self-efficacy and whether patient activation serves as the mediation parameter playing a regulatory role require additional research. Table 2 shows that dimensions – “participation in treatment” and “enriching knowledge” gained the lowest scores indicating that it is urgent to provide participants with high-quality health education related to disease knowledge. In addition, it is necessary to guide them to build awareness of participating in treatment. Enriching knowledge is a generally accepted influencing factor in transforming the coping strategy of patients, which means that the more patients know about their disease and health condition, the easier they would put the disease in perspective and accept reality. Participation in treatment is a growing trend in the global healthcare area. Supplying patients with more chances of taking part in treatment by improving cognition and promoting decision-making concerning the disease, relieves their negative emotions in order to enhance their sense of control and change their negative attitude. The finding of this research implies that it is considered to increase the medical staff’s capacity to empower patients by means of senior-friendly improvement in health education models and content. It can make the medical experience of older chronic disease inpatients better and enhance the effect of health education. Consequently, patients would get a high level of health empowerment and patient activation.

## 5 Conclusion

This study showed that older patients with chronic disease had a middle level of patient activation, which was primarily related to whether they have religion, family *per capita* monthly income, health empowerment, and e-health literacy. It is suggested that medical staff should improve the health education model to help patients advance their e-health literacy level and encourage patients to participate in healthcare decision-making through enhancing health empowerment to boost patient activation and enhance their self-health management ability.

## 6 Limitations

The research sites selected in this study are relatively limited due to external conditions, including time and space constraints. Only one Class A tertiary hospital in Zhengzhou was selected. At the same time, the convenience sample used in this research may limit the generalizability of results. It is recommended to carry out large-scale, multi-center collaborative research in the future to make the research results more representative. In addition, the comorbidities and severity of chronic disease were not considered influencing factors which may had a confounder effect. Therefore, longitudinal studies or qualitative studies can be attempted in order to describe the current status comprehensively and influencing factors of the patient activation of older patients with chronic disease to provide a reference for the comprehensive enhancement of the self-health management ability of older patients with chronic illnesses.

## Data availability statement

The original contributions presented in the study are included in the article/Supplementary material, further inquiries can be directed to the corresponding author.

## Ethics statement

The studies involving humans were approved by Zhengzhou University Life Science Ethics Review Committee. The studies were conducted in accordance with the local legislation and institutional requirements. The participants provided their written informed consent to participate in this study.

## Author contributions

ZYi: Data curation, Formal analysis, Writing – original draft, Conceptualization, Investigation. ZYa: Writing – review & editing, Conceptualization, Funding acquisition, Methodology, Resources. XL: Writing – review & editing. LX: Writing – review & editing. ZX: Writing – review & editing, Polishing and modifying. TY: Writing – review & editing, Conceptualization.
