# Supplementary material for: Current status and influencing factors of activation of older patients with chronic disease
Source: Front Public Health. 2024 Jan 24;11:1308196. doi: 10.3389/fpubh.2023.1308196 (PMC10847314; doi:10.3389/fpubh.2023.1308196)
Supplement: Supplementary file 1 [file Data_Sheet_1.PDF]

DeepL

Subscribe to DeepL Pro to edit this document.

No. _________

Dear Grandma and Grandpa:

Hello! This is a study on "A study on the current status and factors influencing the motivation of elderly inpatients with chronic diseases

"We kindly ask you to answer the questionnaire based on your current situation, there are no right or wrong answers in this questionnaire and it is intended for academic research only. We hope that your answers will help us to understand something about your situation and provide baseline information for the next stage of mentoring work to be carried out by the medical staff concerned. This questionnaire is anonymous, has 4 sections in total, takes approximately 15-20 minutes to complete and is returned on site.

Your personal information will not be disclosed in any of the research reports. Thank you for your great assistance!

Here's to: good health for you and your family!

(Note: Please tick“√”directly on the serial number or fill in the appropriate text on the"___")

I. General information questionnaire and Clinical Data

1. **Your gender:** ☐ Male ☐ Female
2. **Your age:** ☐ < 65 years old ☐ ≥ 65 years old
3. **Are you religious:** ☐ Yes ☐ No
4. **Your permanent place of residence:** ☐ Rural ☐ Town ☐ Urban
5. **Your living arrangement:** ☐ Living alone (or in an institution) ☐ Live together as husband and wife ☐ Cohabitation with children
6. **Number of children:** ☐ ≤ 1 ☐ > 1
7. **Educational level:** ☐ College and above ☐ High school/technical school/secondary school ☐ Junior high school ☐ Primary and below
8. **Previous Occupation:** ☐ Worker ☐ Farmer ☐ Medical Worker ☐ Teacher ☐ Civil Servant or Business Employee ☐ Else
9. **Longest duration of a chronic disease:** ☐ <1 year ☐ 1 to 5 years ☐ 6 to 10 years ☐ >10 years
10. **Duration of this hospital stay:** ☐ Multi-course patients ☐ 3 to 7 days ☐ 8 to 14 days ☐ >14 days
11. **Had a chaperone during hospitalisation:** ☐ Always ☐ Occasionally ☐ Never
12. **Whether they initiate discussions with patients about their condition:** ☐ Volunteer ☐ Not initiated, but will participate ☐ Non-participation
13. **Are multiple chronic disease patients:** ☐ Yes ☐ No
14. **Your medical payment method:** ☐ Urban workers' medical insurance ☐ Medical insurance for urban and rural residents ☐ NACS ☐ Else
15. **Department:** ☐ Nephrology ☐ Cardiology ☐ Oncology ☐ Endocrinology ☐ Respiratory Medicine ☐ Gastroenterology

II. Patient Activation Measure

Entry (in a dictionary, encyclopedia etc)	Strongly disagree.	disagree	agree with	couldn't agree more	inapplicable
1.When all is said and done, I am the person who is responsible for managing my health condition.	1	2	3	4	0
2. Taking an active role in my own health care is the most important factor in determining my health and ability to function.	1	2	3	4	0
3. I am confident that I can take actions that will help prevent or minimize some symptoms or problems associated with my health condition.	1	2	3	4	0
4. I know that every prescription drug prescribed by a physician.	1	2	3	4	0
5. I am confident that I can tell when I need to go get medical care and when I can handle a health problem myself.	1	2	3	4	0
6. I am confident I can tell my health care provider concerns I have even when he or she does not ask.	1	2	3	4	0
7. I am confident that I can follow through on medical treatments I need to do at home.	1	2	3	4	0
8. I understand the source of the problems and origins of my health problems.	1	2	3	4	0
9. I know that I have different treatment options for my health.	1	2	3	4	0
10. I have been able to maintain the lifestyle changes for my health that I have made.	1	2	3	4	0
11. I know how to prevent further problems with my health condition.	1	2	3	4	0
12. I am confident that I will find a solution to my health issues when I confront new situations and problems.	1	2	3	4	0
13. I am confident that I can maintain lifestyle changes like diet and exercise even during times of stress.	1	2	3	4	0

II. Chinese version of e-Health Literacy Scale

entry (in a dictionary, encyclopedia etc)	Very much not.	not matching up	unclear	basically in line with	tallying with
1. I know how to find helpful health resources on the Internet.	1	2	3	4	5
2. I know how to use the Internet to answer my health questions.	1	2	3	4	5
3. I know what health resources are available on the Internet.	1	2	3	4	5
4. I know where to find helpful health resources on the Internet.	1	2	3	4	5
5. I know how to use the health information I find on the Internet to help me.	1	2	3	4	5
6. I have the skills I need to evaluate the health resources I find on the Internet.	1	2	3	4	5
7. I am able to differentiate between high and low quality health information on the Internet.	1	2	3	4	5
8. I feel confident in using information from the Internet to make health decisions.	1	2	3	4	5

IV. Health Empowerment Scale for Elderly with Chronic Disease

sports event	couldn't agree more	agree with	usual	disagree	Strongly disagree.
1. I'm responsible for taking care of myself.	1	2	3	4	5
2. I have the confidence to take care of myself.	1	2	3	4	5
3. I have a responsibility to take care of my loved ones.	1	2	3	4	5
4. I have the confidence to take care of my loved ones.	1	2	3	4	5
5. I can get support and care from my family.	1	2	3	4	5
6. I could use the support and help of my friends.	1	2	3	4	5
7. It's easy for me to get to the doctor.	1	2	3	4	5
8. I have access to good medical care.	1	2	3	4	5
9. I am active in group activities.	1	2	3	4	5
10. I will ask for help when I can't solve a problem on my own.	1	2	3	4	5
11. I'm active in learning about the disease.	1	2	3	4	5
12. I know where to get knowledge about the disease.	1	2	3	4	5
13. I know what causes disease.	1	2	3	4	5
14. I know what the test results mean.	1	2	3	4	5
15. I know how to prevent the disease from progressing further.	1	2	3	4	5
16. I take an active interest in health and wellness.	1	2	3	4	5
17. I am active in disease prevention and control.	1	2	3	4	5
18. I participate in the development of treatment programs..	1	2	3	4	5
19. I will talk to my healthcare provider if the treatment plan is not right for me.	1	2	3	4	5
20. I always monitor the disease myself.	1	2	3	4	5
21. I've become more conscious of eating healthy since I got sick.	1	2	3	4	5
22. I can deal with the negative emotions of my illness.	1	2	3	4	5
23. I was able to face the disease with optimism and ease of mind.	1	2	3	4	5
24. I'm content with my life as it is.	1	2	3	4	5
25. Even when I'm sick, I still have hope in life.	1	2	3	4	5

26. I've adapted to living with an illness.	1	2	3	4	5

Completion of visit time:
